# SIADH and Lung Small Cell Carcinoma: A Clinical Case

**DOI:** 10.7759/cureus.97425

**Published:** 2025-11-21

**Authors:** Nuno Oliveira, Francisco San Martin, Rosa Amorim

**Affiliations:** 1 Internal Medicine, Unidade Local de Saúde do Oeste - Unidade Caldas da Rainha, Caldas da Rainha, PRT

**Keywords:** differential diagnosis of hyponatremia, siadh of malignancy, small-cell lung carcinoma, symptomatic hyponatremia, syndrome of inappropriate secretion of antidiuretic hormone (siadh)

## Abstract

Hyponatremia is the most common electrolyte disorder found in clinical practice. One of the causes is the syndrome of inappropriate antidiuretic hormone secretion (SIADH). SIADH is characterized by hypotonic and euvolemic hyponatremia with elevated urinary osmolarity and increased ADH secretion without a triggering stimulus. This pathology is often associated with neoplastic conditions, namely lung neoplasms.

We report the case of a 54-year-old woman admitted with a history of vertigo and uncontrollable nausea with hyponatremia of 100meq/L at admission and diagnosed with paraneoplastic SIADH secondary to small cell lung carcinoma. After establishing the diagnosis, the patient was transferred to the Portuguese Oncology Institute of Lisbon (IPO), where she began chemotherapy.

This case highlights the challenge of controlling sodium levels before initiating oncologic treatment and illustrates how chronic hyponatremia may allow for some degree of neurological adaptation to markedly low sodium concentrations.

## Introduction

Hyponatremia is a very common hydroelectrolytic disorder. It is defined as a plasma sodium concentration <135 mEq/L (with slight variation depending on laboratory reference values), meaning a level below the laboratory’s lower cut-off. Importantly, the presence of hyponatremia does not necessarily reflect the patient’s hydration status. It can occur in all three volume states: hypovolemia, euvolemia, and hypervolemia [1,2].

Thus, first of all, hyponatremia must be objectified, as pseudo-hyponatremia, in states of plasma hyperglycemia or hypertriglyceridemia, can falsify the sodium value. The following formula can be used:

$\text{Corrected Sodium} = \text{Measured Sodium} + 1.6 \times \left( \frac{\text{Glucose}}{100} \right)$

Once true hyponatremia is confirmed, it is important to interpret plasma sodium, not as an absolute quantity but as an ion dissolved in a liquid medium. Hyponatremia, therefore, reflects a change in sodium concentration relative to water. In hypervolemic and euvolemic states, the problem is not a deficiency of sodium but an excess of water, which drastically alters the therapeutic strategy.* *Even in hypovolemic hyponatremia, there is a relative excess of water compared with sodium, although both still require correction [1].* *

In a hypovolemic state, patients present with dry mucous membranes, positive skin turgor, and tachycardia with decubitus hypotension or orthostatic hypotension. This reflects accentuated cutaneous, renal, or gastrointestinal losses, with very concentrated urine and low excreted sodium, indicating compensatory renal sodium and water retention [1-3].

In the hypervolemic state, patients present with localized or generalized (anasarca) edema. This alteration can arise with multiple pathologies, namely heart failure or ascites in liver cirrhosis. These patients exhibit compensatory renal responses, producing a small volume of concentrated urine with low sodium content. In this situation, there is no true volume depletion; instead, fluids become functionally sequestered in a third space, leading to a reduction in circulating volume [1-3].

In the euvolemic state, the scenario is more complex. Patients show no signs of dehydration and no evidence of edema. Urine may be either concentrated or diluted; in cases where it is markedly diluted, excessive fluid intake, such as in primary polydipsia or potomania, is likely. In this setting, urinary sodium levels are typically elevated [1-3].

Syndrome of inappropriate antidiuretic hormone secretion (SIADH) is a cause of hypotonic and euvolemic hyponatremia with elevated urinary osmolarity and increased ADH secretion without a triggering stimulus. The term was created in 1957 when Schwartz et al described the inability of the kidney to reabsorb sodium in patients with lung carcinoma [2-4].

Antidiuretic hormone (ADH), or arginine vasopressin, is synthesized in the supraoptic and paraventricular nuclei of the hypothalamus and stored in the pituitary gland. It is then secreted in response to osmotic and non-osmotic stimuli. Osmotic stimuli are determined by an increase in plasma osmolarity (above 284 mOsm/Kg). Non-osmotic stimuli are in response to the stimulation of nociceptive pathways, vomiting, medications, or hypoglycemia. ADH acts on V1 receptors located in the arterial wall, causing smooth muscle contraction and an increase in blood pressure. It acts on V3 receptors in the pituitary gland, stimulating the release of ACTH. Finally, it acts on receptors in the cells of the renal collecting ducts, increasing the expression of aquaporin-2 and promoting the reabsorption of free water [1,2].

Thus, the pathophysiology of SIADH is based on the increase in the amount of ADH secreted, either due to increased production in response to a non-stimulus or an ectopic source of ADH. In paraneoplastic conditions, this increase in ADH is believed to arise from an ectopic source. Nevertheless, it does not relate to tumor size or the presence of metastases. However, recent laboratory findings have reported cases in which the plasma level of ADH is normalized. In these cases, the pathology results from a constitutive activation of V2 receptors, with an increase in aquaporins even under normal ADH [2,3].

Lung neoplasms, particularly small cell carcinoma, are very frequently associated with SIADH. Approximately 7-16% of SIADH cases are caused by small cell lung carcinoma (SCLC), which accounts for about 70% of all paraneoplastic SIADH cases [2-6].

## Case presentation

A 54-year-old woman presented to the Emergency Department with a 3-week history of dizziness, vertigo, and headaches, accompanied by nausea and vomiting. The vomiting started in the week after the start of her holidays, with no family epidemiological context, about 5-10 minutes after a meal, with no relation to the type of food and no associated abdominal pain. The vomitus was food content, without blood or other characteristics. The patient reported liquid stools, without colic, only between 5 and 8 days. She had 2 daily episodes of liquid stools without blood, mucus, or pus, and the condition resolved spontaneously. The headache was holocranial, without afternoon/morning predominance, and without a triggering or relieving factor. She also reported a long-standing dry cough that had worsened over the past 8 days, which prompted her to visit her family doctor, who prescribed amoxicillin-clavulanic acid, acetylcysteine, prednisolone, salmeterol with fluticasone, and betahistine. She denied chills, fever, faintness, blurred vision, blood loss, polydipsia, polyphagia, polyuria, night sweats, weight loss, or other symptoms. The patient had a smoking history of 18 pack-years, and her usual medications are listed in Table 1.

**Table 1:** Patient's usual prescribed medications

Usual Medication	
Clebopride, 0.5 mg	1 pill at breakfast
Perindopril + Indapamide, 5 mg + 1.25 mg	1 pill at breakfast
Beta-histine, 24 mg (2 weeks before admission)	1 pill every 12 hours
Prednisolone 10 mg (some days before admission)	1 pill at lunch

Upon admission, she was hypertensive at 153/86 mmHg, normocardic, afebrile, without leg edema, and had coarse breath sounds in both hemithoraces. The major analytical findings are summarized in Table 2.

**Table 2:** Relevant laboratory values

Parameter	Measured value	Reference value
Sodium (Na)	100 meq/L	135-145 meq/L
C-Reactive Protein (CRP)	7 µg/dL	<0,5 µg/dl
Leukocytes	14800 cells/mL	4000-10000 cells/mL

**Figure 1:**
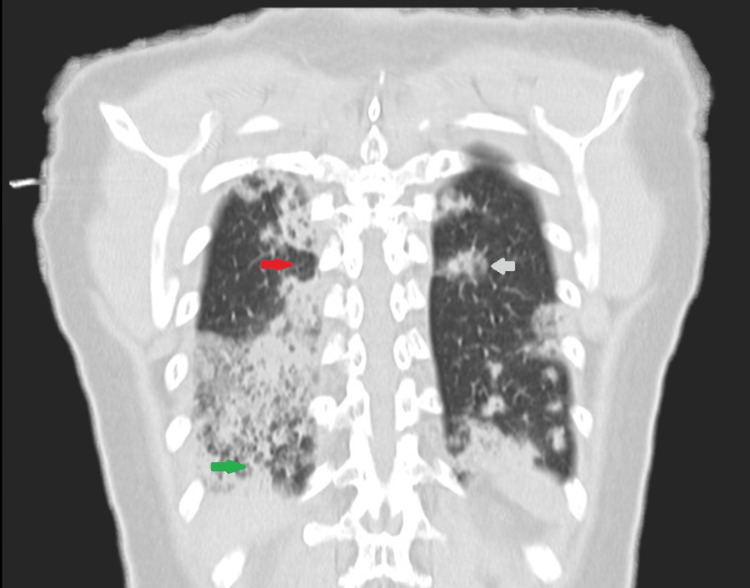
Thoracic CT-scan Red arrow shows enfisema bubbles; green arrow shows a mosaic pattern; blue arrow shows a nodular formation with soft-tissue density (impacted mucous vs adenomegaly)

**Figure 2:**
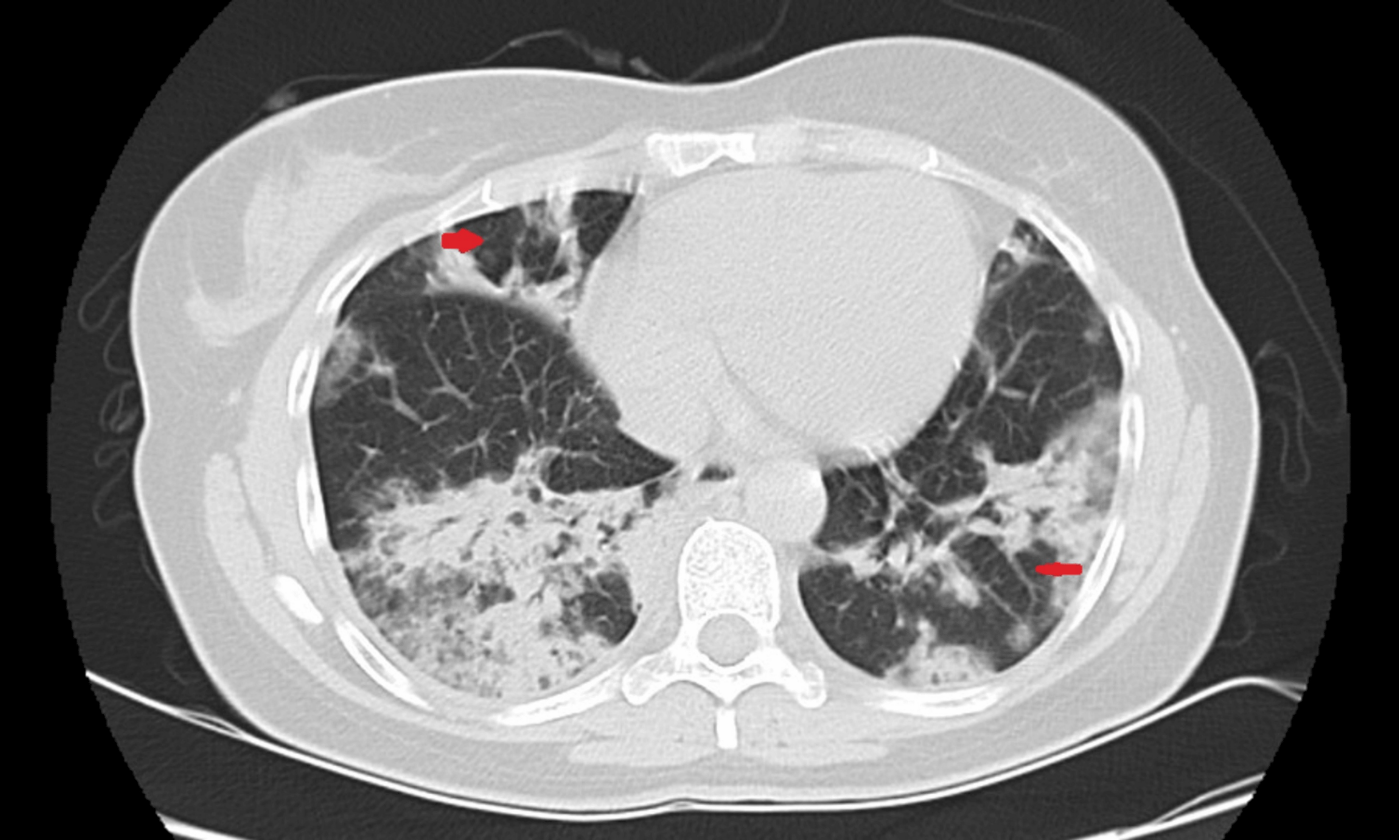
Thoracic CT-scan Red arrow indicates emphysema bubbles

**Figure 3:**
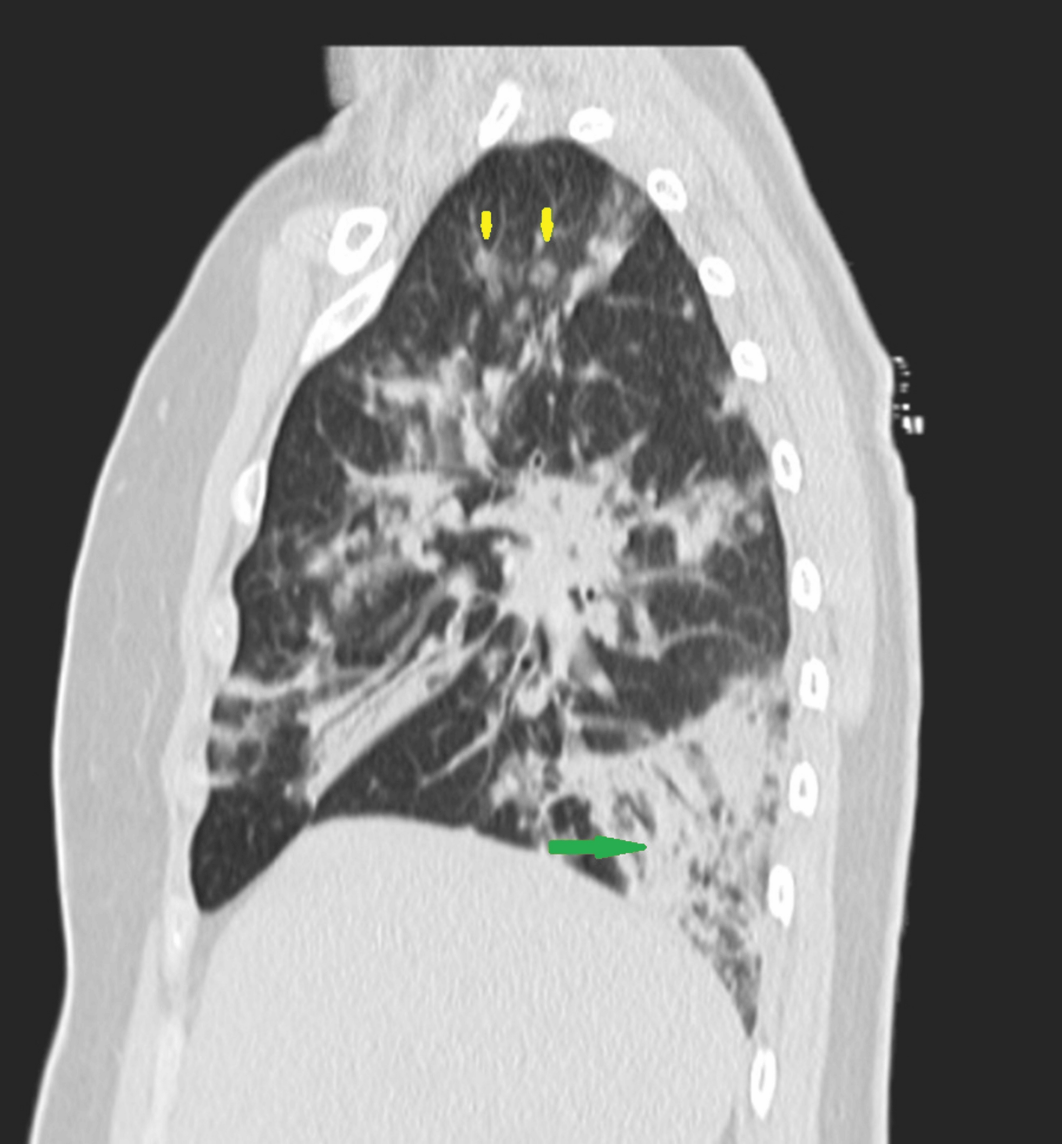
Thoracic CT-scan Green arrow indicates a mosaic/crazy paving pattern; yellow arrows indicate nodular neoplastic lesions

The hyponatremia was accompanied by hypoosmolarity, and correction of the sodium level was initiated. The possibility of hyponatremia due to vomiting, an iatrogenic effect of indapamide, or another cause had yet to be clarified. As the dizziness did not respond to betahistine, a contrast-enhanced head CT scan was requested, which showed no abnormalities. Due to suspicion of condensations on the X-ray, a thoraco-abdomino-pelvic CT scan (Figures 1-3) was requested. The CT scan showed paraseptal emphysema in the upper lobes and areas of ground-glass opacity in the lower lobes, middle lobe, and lingula, with small foci of mucoid impaction in the right lower lobe, suggesting inflammatory change. Diffuse thickening of bronchial walls and rounded formations with soft tissue density in the right peri-hilar region were observed, raising suspicion for bronchiectasis with content or adenomegaly. The lymph node in the right paratracheal region appears to have dimensions within normal limits. The right lower lobe was found to have an indeterminate nodular image and areas of ground-glass opacities in a mosaic pattern.

**Figure 4:**
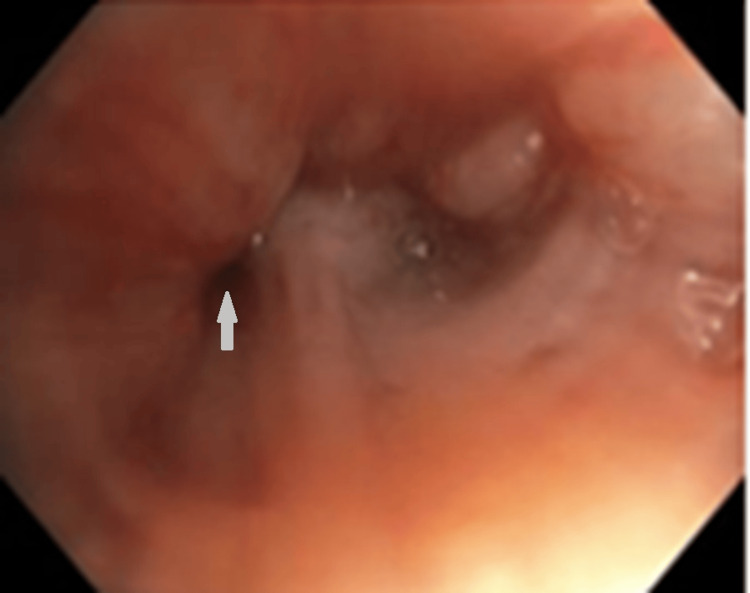
Bronchofibroscopy of the right bronchi showing an extreme narrow airway Grey arrow shows extreme airway limitation

The case was discussed with Pulmonology, who, after reviewing the imaging, noted the presence of crazy-paving patterns and recommended performing a bronchoscopy. Bronchofibroscopy (BFC) revealed an infiltrated and hypervascularized mucosa in the posterior segment of the right lower lobe, along with narrowing of the entrance orifice (Figure 4). 

During the bronchofibroscopy, saline lavage was performed, and secretions were aspirated. The collected samples were sent for cytomorphological, bacteriological, and mycobacteriological analyses, as well as testing for SARS-CoV-2 and *Pneumocystis jirovecii.* During this period, the patient’s hyponatremia was corrected gradually using a 3% NaCl solution, achieving an increase of 3-5 mEq/L per day. After serum sodium levels normalized, an attempt was made to discontinue fluid therapy. The patient was maintained on oral sodium supplementation; however, within eight hours, the sodium level dropped by approximately 8 mEq/L, prompting the resumption of intravenous supplementation.

While awaiting the results of the BFC samples, additional laboratory studies were requested (Table 3).

**Table 3:** Additional laboratory findings

Lab item	Measured value	Reference value
Cortisol/Creatinine ratio	7 µg/g creatinine	0,7-85 µg/g creatinine
Urinary Na 24h	46 mmol/L	40-220 mmol/L
HIV 1/2	Negative	n/a
Angiotensin-converting enzyme (ACE)	16 U/L	8-52 U/L
Adrenocorticotropic hormone (ACTH)	16.5 ng/mL	7-63,3 ng/mL

The hypothesis of paraneoplastic SIADH secondary to a probable lung neoplasm was then raised, and among the samples collected during the BFC, the biopsy result was notable for identifying SCLC (AE1/AE3^+^, TTF1^+^, CD56^+^, Synaptophysin^+^, focal expression of Chromogranin A, and Ki-67 at 90%).

Given the results obtained, the case was again discussed with the Pulmonology Department of our hospital center, which recommended discharge with referral to Medical Oncology. Thus, given the local limitations, we contacted the Portuguese Oncology Institute (IPO). The patient could not be discharged because of the severity of her hyponatremia. However, colleagues at the IPO agreed to accept her for further management. A positron emission tomography (PET) scan showed evidence of metabolically active disease in the lungs, lymph nodes, and bones. She began a 3-week chemotherapy program with the Etoposide/Cisplatin (EP) regimen on a 21-day cycle. She is currently being followed up at the IPO.

## Discussion

In this clinical case, given the markedly low sodium level at admission and absence of altered consciousness, a chronic onset of hyponatremia was considered highly likely, although no recent laboratory results were available to confirm this. Euvolemic hyponatremia with decreased plasma osmolarity was suspected. Assuming a chronic presentation, we considered the possibility of an iatrogenic effect from long-term therapy with Indapamide [7-12]. There was also the possibility of iatrogenic hyponatremia due to prednisolone; however, as it had been prescribed only three days earlier, this was considered unlikely. On the other hand, the presence of vomiting would be either an etiological possibility or a consequence of hyponatremia at that time [10-13]. With the start of intravenous correction and suspension of probable etiological agents, serum sodium levels slowly normalized. However, as soon as intravenous therapy was suspended, a sharp drop in natremia was recorded. At the beginning of hospitalization, the patient continued to experience vomiting and dizziness, which were unresponsive to betahistine and occurred despite a normal CT scan; these symptoms resolved after normalization of serum sodium. In fact, even under strict fluid restriction of approximately 500 mL per day, a decline in sodium levels was consistently observed, requiring intravenous therapy to correct the natremia. Despite maintaining fluid restriction and administering oral salt supplementation (approximately 6-9 g of additional salt daily), the sodium levels continued to fall.

This case reinforces the link between small cell carcinoma and SIADH [8,10,12,13]. However, several confounding factors were present from the outset, including vomiting, chronic diuretic therapy, a short course of corticosteroids of about one week, and the absence of constitutional symptoms. The vertigo symptoms, for example, were a confounding factor, raising the question of whether they were due to the neurological effects of hyponatremia, a paraneoplastic manifestation of SCLC, or a combination of both [8,9,12]. These aspects needed to be carefully analyzed and contextualized within this clinical scenario. The persisting low sodium levels were perhaps our strongest indication of a metabolic cause, given the ongoing decline. The continued drop, despite the removal of potential offending agents, clearly indicates an active underlying cause [8,12]. 

In summary, the persistent and challenging refractoriness of the hyponatremia to standard management, including the slow normalization with intravenous correction and the rapid relapse despite fluid restriction and high oral salt supplementation, provided the strongest evidence of an active underlying metabolic disorder. While initial confounding factors such as medication iatrogenesis (indapamide, prednisolone) and non-specific symptoms (vomiting, vertigo) obscured the immediate diagnosis, the clinical trajectory ultimately reinforced the strong, known association between SCLC and SIADH. This case, therefore, highlights the critical need for a high index of suspicion for paraneoplastic SIADH in SCLC, even when the initial presentation is complicated by multiple potential contributing factors. 

## Conclusions

This case underscores the importance of prioritizing patient care above all. At times, this requires reassessing or challenging existing clinical opinions. The patient could not have been safely discharged without initiating chemotherapy or other targeted therapy for the underlying neoplasm, which prompted referral to the IPO. Normalization of serum sodium was achieved only after commencement of chemotherapy.

Despite the challenges in diagnosing SIADH, a systematic and methodical evaluation of the different aspects of hyponatremia allowed us to reach a good diagnosis. Moreover, this case demonstrates that sustained normalization of serum electrolytes can be achieved only by addressing the underlying cause of SIADH.

## References

[1] Rocha PN (2011). Hyponatremia: basic concepts and practical approach (Article in Portuguese). J Bras Nefrol.

[2] Mentrasti G, Scortichini L, Torniai M, Giampieri R, Morgese F, Rinaldi S, Berardi R (2020). Syndrome of inappropriate antidiuretic hormone secretion (SIADH): optimal management. Ther Clin Risk Manag.

[3] Kanaji N, Watanabe N, Kita N (2014). Paraneoplastic syndromes associated with lung cancer. World J Clin Oncol.

[4] Schwartz WB, Bennett W, Curelop S, Bartter FC (1957). A syndrome of renal sodium loss and hyponatremia probably resulting from inappropriate secretion of antidiuretic hormone. Am J Med.

[5] Berardi R, Santoni M, Rinaldi S (2016). Risk of hyponatraemia in cancer patients treated with targeted therapies: a systematic review and meta-analysis of clinical trials. PLoS One.

[6] Onitilo AA, Kio E, Doi SA (2007). Tumor-related hyponatremia. Clin Med Res.

[7] Adrogué HJ, Tucker BM, Madias NE (2022). Diagnosis and management of hyponatremia: a review. JAMA.

[8] Ghosal A, Qadeer HA, Nekkanti SK, Pradhan P, Okoye C, Waqar D (2023). A conspectus of euvolemic hyponatremia, its various etiologies, and treatment modalities: a comprehensive review of the literature. Cureus.

[9] Grant P, Ayuk J, Bouloux PM (2015). The diagnosis and management of inpatient hyponatraemia and SIADH. Eur J Clin Invest.

[10] Sumi H, Tominaga N, Fujita Y, Verbalis JG (2025). Treatment of hyponatremia: comprehension and best clinical practice. Clin Exp Nephrol.

[11] Barahman M, Shamsaei G, Kashipazha D, Bahadoram M, Akade E (2024). Paraneoplastic neurological syndromes of small cell lung cancer. Postep Psychiatr Neurol.

[12] Garrahy A, Thompson CJ (2017). Syndrome of inappropriate antidiuresis should it be managed by specialised endocrinologists?. Endocrine.

[13] Warren AM, Grossmann M, Christ-Crain M, Russell N (2023). Syndrome of inappropriate antidiuresis: from pathophysiology to management. Endocr Rev.

